# Detection of pathogens within Ixodid ticks collected from domestic cats across the USA

**DOI:** 10.1186/s13071-025-06902-z

**Published:** 2025-07-04

**Authors:** Rachel C. Smith, Kellee D. Sundstrom, Ruth C. Scimeca, Lindsay A. Starkey

**Affiliations:** Department of Pathobiology, College of Veterinary Medicine, Oklahoma State University, Stillwater, OK USA

**Keywords:** Ixodid ticks, *Borrelia burgdorferi*, *Anaplasma phagocytophilum*, *Cytauxzoon felis*, *Ehrlichia* spp., *Rickettsia* spp., Domestic cats, USA

## Abstract

**Background:**

Ixodid ticks and tick-borne diseases continue to be an emerging health concern in the USA. Companion animals dwell in close proximity with people; therefore, it is important to understand how they might contribute to the maintenance of tick-borne pathogens, especially zoonoses, in the peri-domestic environment. Domestic cats are often overlooked in epidemiological investigations of tick-borne infections compared with their canine counterparts.

**Methods:**

The purpose of this study was to investigate the potential exposure of domestic cats to tick-borne pathogens by molecularly testing adult Ixodid ticks collected from cats that were presented for veterinary care. A total of 802 ticks collected from 512 individual cats were tested by conventional polymerase chain reaction (PCR). Ticks were morphologically identified as *Ixodes scapularis* (*n* = 431), *Amblyomma americanum* (*n* = 218), and *Dermacentor variabilis* (*n* = 153).

**Results:**

The most prevalent pathogen detected was *Borrelia burgdorferi* s.s., detected in 19.5% of *I. scapularis*. *Ehrlichia ewingii* was detected in 3.2% of *A. americanum*. *Anaplasma phagocytophilum* was detected in 0.7% of *I. scapularis*. *Cytauxzoon felis* was detected in 0.5% of *A. americanum*. *Borrelia miyamotoi* was detected in 0.2% of *I. scapularis*. Submitting clinics were contacted to gather additional information on cats infested by pathogen-infected ticks. This information did not yield a meaningful relationship between potential pathogen exposure and development of clinical signs around the time of tick collection.

**Conclusions:**

This study is the largest survey for pathogens within Ixodid ticks collected from domestic cats in the USA and the only survey in which retrospective clinical information was retrieved. While the effect of many tick-borne pathogens on feline health remains unclear, this study demonstrates that cats infested with ticks are at risk for pathogen exposure and may be a source for harboring pathogen-infected ticks in and around the home.

**Graphical Abstract:**

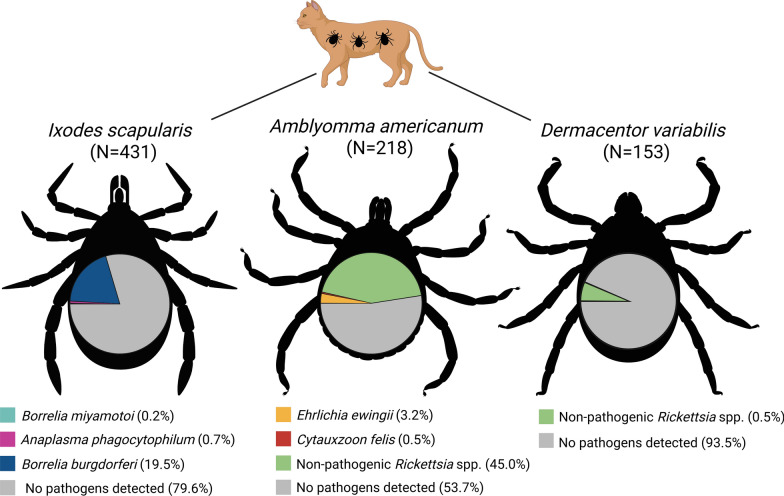

**Supplementary Information:**

The online version contains supplementary material available at 10.1186/s13071-025-06902-z.

## Background

In the USA, ticks and tick-borne pathogens persist as significant and growing threats to public and veterinary health [1]. The tick species *Amblyomma* spp., *Dermacentor variabilis*, and *Ixodes scapularis* are among those that pose the greatest risk to human and animal health and continue to expand their geographic distributions as a result of changing climatic conditions, land use, and movement of humans and animals [2]. Companion animals live in close proximity to humans; therefore, it is important to consider their role in pathogen transmission through vectors [3, 4]. The importance of tick-borne infections in dogs is well recognized and continues to be the focus of prevalence studies in companion animals. In tick surveys conducted in the USA with both dogs and cats represented, dogs are more frequently parasitized by ticks than cats and appear to sustain higher intensity infestations [5, 6]. Dogs are capable of maintaining some tick-borne zoonoses for extended periods of time, with or without clinical signs, and, thus, act as reservoirs [7–9]. Furthermore, dogs may be useful sentinels for estimating risk of human infection for some tick-borne agents within the same geographic area [10–13]. In cats, the frequency and clinical significance of tick-borne infections of human and animal importance, including *Anaplasma* spp., *Ehrlichia* spp., and *Borrelia burgdorferi*, remain unclear [14]. Several reports of nonspecific clinical signs in cats naturally infected with these pathogens have been previously published, [15–20]; however, similar clinical manifestations have not been reproducible following experimental infection [21].

Investigation into the prevalence of tick parasitism and exposure to tick-borne pathogens is limited among domestic cats, with only few broad geographic distribution surveys in the USA published to date [17, 22, 23]. There is, however, a well-characterized *Cytauxzoon felis* distribution knowledge in cats [24]. Difficulty in acquiring a large and geographically diverse collection of feline blood samples and the lack of commercial in-clinic diagnostic assays approved for use in cats limit carrying out robust prevalence surveys for tick-borne agents. One approach to estimating exposure of hosts to tick-borne agents while minimizing these barriers is to collect ticks directly from the host population and molecularly test the ticks for the presence of disease agents. This style of epidemiologic survey has previously been conducted in both cats and dogs [22, 25, 26]. The purpose of the present study was to evaluate Ixodid ticks collected from domestic cats by veterinary clinics across the USA for the presence of tick-borne pathogens. The target pathogens tested were *Anaplasma phagocytophilum*, *Borrelia* spp., *Cytauxzoon felis*, *Ehrlichia* spp., and *Rickettsia* spp., all of which are zoonotic with the exception of *C. felis*. When pathogen-infected ticks were identified, the submitting veterinary clinics were contacted to gather additional clinical and contextual data on the infested cats.

## Methods

### Specimen acquisition and selection

The ticks used in this study were selected from banked specimens that were collected through the national Show Us Your Ticks survey (https://www.showusyourticks.org/), which is elsewhere described [6]. To briefly summarize the survey protocol: each survey submission contained ticks collected from a singular host at their veterinary clinic, which were mailed to the Oklahoma State University College of Veterinary Medicine. Upon original receipt, ticks were morphologically identified using standard dichotomous keys and a dissecting microscope [27, 28]. Ticks were then stored in 70% ethanol at −20 °C and remained under these conditions until use in the present study. Tick specimens were included from the survey collection for this study if they met the following criteria: (i) they were collected from a domestic cat, (ii) they were adult-stage Ixodid ticks, (iii) they were collected between January 2019 and December 2023, and (iv) they had not been previously utilized for DNA extraction or pathogen detection. When multiple ticks were available from the same cat, all ticks meeting the selection criteria were utilized.

### Nucleic acid extraction and pathogen detection

Prior to dissection and nucleic acid extraction, the sex, life stage, and morphological identification of each selected tick was confirmed. The excess ethanol was dried from ticks using a Kimwipe, ticks were individually dissected, and genomic DNA was extracted from the complete internal contents of each tick, using the Cytiva Blood genomicPrep Mini Spin Kit (Cytiva, Marlborough, MA, USA) according to the manufacturer’s protocol. A template-free negative control was extracted alongside each batch of tick specimens. Extracted DNA was stored at −80 °C for further analysis. All pathogen detection was carried out by nested conventional polymerase chain reaction (PCR). Ticks were tested for a subset of tick-borne pathogens on the basis of their morphological identity and corresponding to well-described vector–pathogen relationships. Testing of *I. scapularis* utilized primers for *Borrelia* spp. [29] and *A. phagocytophilum* [30]; *A. americanum* was tested for *Ehrlichia ewingii*, *Ehrlichia chaffeensis* [31, 32], *C. felis* [33, 34], and *Rickettsia* spp. [35, 36]; and *D. variabilis* was tested for *C. felis* and *Rickettsia* spp. (Supplementary Table S1).

Thermocycling conditions and reaction mixtures were carried out as previously described [22]. All PCR reactions were performed alongside a known positive control, a template-free water control, and the template-free extraction control corresponding to each batch of samples. Amplicons were resolved by electrophoresis on 2.0% agarose gel with GelRed^®^ staining (Biotum, Fremont, CA, USA). Amplicons matching the size of the positive control were purified using the Wizard^®^ SV Gel and PCR Clean-Up System (Promega, Madison, WI, USA) and submitted for Sanger sequencing at the Oklahoma State University Molecular Core Facility (Stillwater, OK, USA). Sequence identity was determined by BLAST comparison against the National Center for Biotechnology Information database, GenBank.

### Retrospective clinical and contextual data collection

For cats harboring pathogen-infected ticks with evidence that blood feeding had occurred (engorgement), their respective clinics were contacted via email address, provided on the original survey submission form. Clinics were requested to answer a brief questionnaire that included included description of patient’s ectoparasite control, time spent outside the house, clinical status, and history of tick-borne disease. Raw responses were collated and summarized categorically.

### Statistical analysis and data visualization

All statistical analyses were performed in R Studio (version 2024.09.1., Boston, MA, USA). Confidence intervals (CI 95%) were calculated for prevalence. A two-proportion *Z*-test was used to evaluate differences in pathogen positivity between male and female ticks, with significance designated at *P* < 0.05. Maps and graphical figures were also constructed using R Studio.

## Results

### Sample distribution and pathogen detection

A total of 802 adult Ixodid ticks were available for inclusion and tested in this study. These consisted of *I. scapularis* (*n* = 431), *A. americanum* (*n* = 218), and *D. variabilis* (*n* = 153). Ticks were collected from 512 individual cats at 148 private veterinary clinics, rescue/shelter organizations, or academic teaching hospitals. Cats were infested with 1–17 adult ticks per cat, with an average of 1.52 ticks per cat. The vast majority of ticks were submitted from the eastern half of the USA, with 36 states represented (Supplementary Fig. S1).

**Fig. 1:**
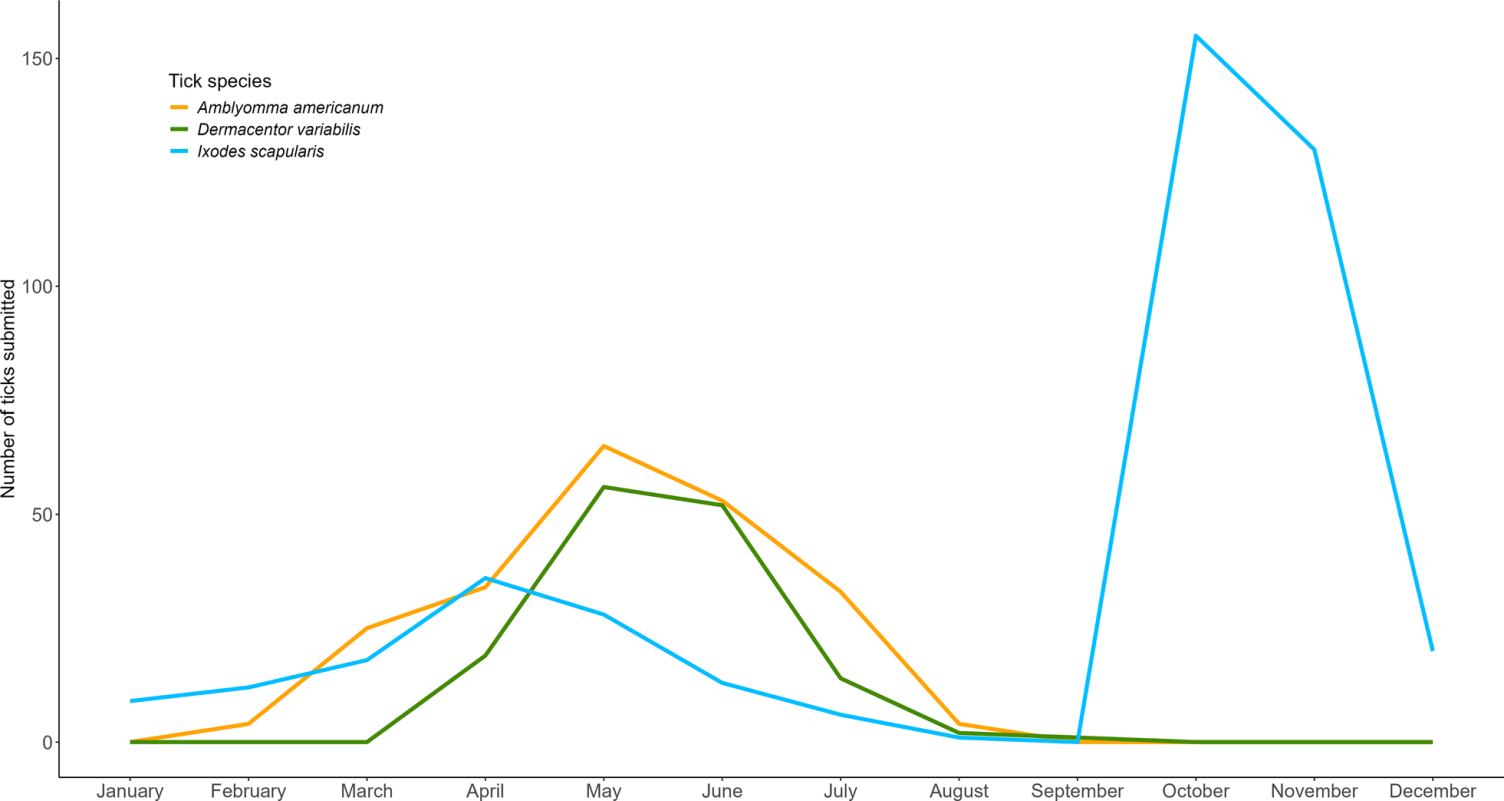
Monthly distribution of Ixodid tick collection from domestic cats

When all tick species and geographies are considered, ticks were collected from cats during every month of the year, although seasonal variation occurred between species as expected (Fig. 1).

Estimated time spent outside for cats included in this study was reported for 440/512 cats. The majority of cats, 66.0% (338/512), reportedly spent ≥ 50% of their time outdoors, and 4.5% (23/512) reportedly spent 0% of their time outdoors. Correlation between time spent outside and number of ticks submitted assessed by Pearson correlation coefficient showed very weak positive linear correlation (*r* = 0.19) between percentage of time spent outside and the number of ticks submitted.

**Fig. 2:**
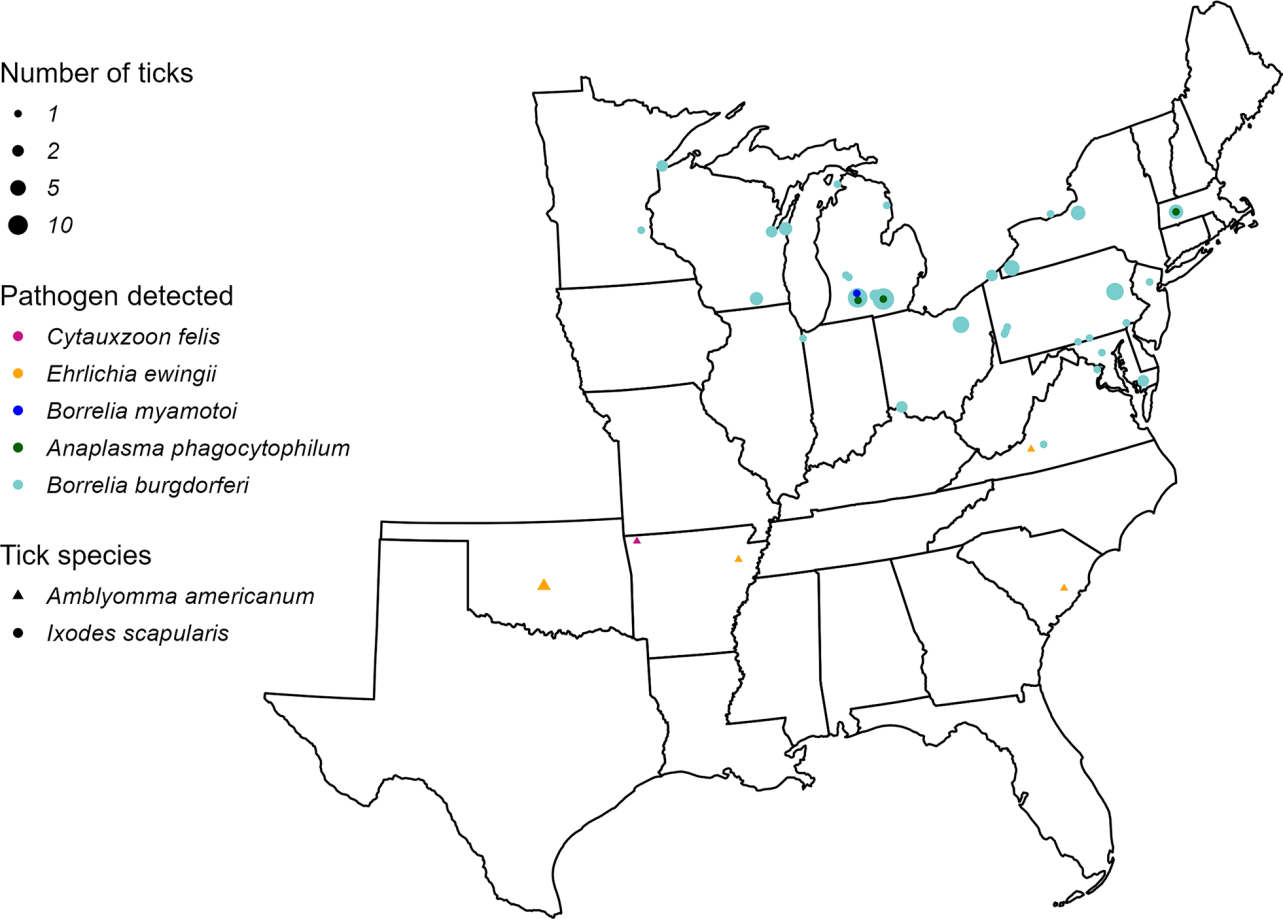
Geographic distribution of pathogen-positive Ixodid ticks collected from domestic cats

In total, 12.0% of tested ticks harbored agents of tick-borne disease (Table 1, Fig. 2).

**Table 1:** Pathogen prevalence by species in Ixodid ticks collected from domestic cats

Tick species	Target pathogen	Prevalence	CI (%)
*Ixodes scapularis* (*n* = 431)	*Borrelia burgdorferi*	19.5% (84/431)	15.9–23.6
	*Borrelia miyamotoi*	0.2% (1/431)	0.0–1.4
	*Anaplasma phagocytophilum*	0.7% (3/431)	0.0–1.4
*Amblyomma americanum* (*n* = 218)	*Ehrlichia ewingii*	3.2% (7/218)	1.4–6.8
	*Ehrlichia chaffeensis*	0%	0.0–2.2
	*Cytauxzoon felis*	0.5% (1/218)	0.0–2.9
	*Pathogenic *Rickettsia* spp.	0%	0.0–2.2
	Nonpathogenic *Rickettsia* spp.	45.0% (98/218)	38.3–51.8
*Dermacentor variabilis* (*n* = 153)	Pathogenic *Rickettsia* spp.	0%	0.0–3.1
	Nonpathogenic *Rickettsia* spp.	6.5% (10/153)	3.4–12.0
	*Cytauxzoon felis*	0%	0.0–3.1
All tick species (*n* = 802)	**Any known pathogen**	12.0% (96/802)	9.8–14.5

^*^Pathogenic *Rickettsia* spp. are any *Rickettsia* spp. that have been commonly reported in association with illness in humans or veterinary species. Nonpathogenic *Rickettsia* spp. include those that have been detected in ticks or mammalian hosts but have not been reported to cause clinical disease

The most prevalent pathogen was *B. burgdorferi*, detected in 19.5% of *I. scapularis* ticks, followed by *E. ewingii* detected in 3.2% of *A. americanum* ticks. *Borrelia miyamotoi*, *C. felis*, and *A. phagocytophilum* were found in less than 1.0% of their respective tick vectors. Neither *E. chaffeensis* nor pathogenic *Rickettsia* spp. were detected in their respective tick vectors. For *A. americanum* and *I. scapularis* a two-sample proportion test was conducted to evaluate difference in pathogen prevalence between females and males of the respective tick species. *Dermacentor variabilis* was excluded from this analysis since no pathogens were detected. No significant difference in pathogen prevalence was observed between females and males of *A. americanum* (*P* = 1.0) or *I. scapularis* (*P* = 1.0). *Rickettsia* spp., which are not known to be pathogenic, were amplified in both *A. americanum* and *D. variabilis*. *Rickettsia* spp. detected in *D. variabilis* included *Rickettsia montanesis* (*n* = 5), *Rickettsia bellii* (*n* = 3), and *Rickettsia rhipicephali* (*n* = 2). *Rickettsia amblyommatis* was detected in *A. americanum* (*n* = 98). Coinfection with *E. ewingii* and *Rickettsia amblyommatis* was observed in *A. americanum* (*n* = 4), but coinfection with two pathogenic agents was not observed in any tick species. Among the surveyed cat population, 15.2% (78/512), or slightly more than 1 in 7 cats, harbored ticks infected with a known pathogen. A small proportion of cats (*n* = 10) were infested by greater than one pathogen-infected tick at a single time point. One cat was infested with two *E. ewingii*-infected *A. americanum*, seven cats were infested with 2–4 *B. burgdorferi*-infected *I. scapularis*, and one cat was infested by four pathogen-infected *I. scapularis*: *I. scapularis* infected with *A. phagocytophilum* (*n* = 1), *I. scapularis* infected with *B. burgdorferi* (*n* = 2), and *I. scapularis* infected with *B. miyamotoi* (*n* = 1). Two cats were infested with pathogen-infected ticks at two different time points. One cat was repeatedly infested with a single *B. burgdorferi*-infected *I. scapularis* 2 weeks apart; one cat was infested with a single *B. burgdorferi*-infected *I. scapularis* followed by infestation with a single *E. ewingii*-infected *A. americanum* approximately 6 months later.

### Cats with pathogen-positive ticks

A total of 80 submissions representing 78 individual cats contained one or more ticks detected as positive for pathogens. Two cats had duplicate submissions, representing two time points on the same individual. Follow-up was only pursued when there was evidence that the pathogen-positive tick fed on the cat, resulting in exclusion of three submissions from which the infected ticks were not engorged and reportedly not attached to the host. Follow-up contact was made via the email address included on the original submission form for the remaining 77 submissions (representing 75 cats) with the following outcomes: responded (*n* = 36), did not respond (*n* = 37), and email delivery failure due to invalid address (*n* = 4). Follow-up responses are categorically summarized (Supplementary Table S2).

As expected, most cats had either no history, known lapses, or suspected lapses of ectoparasite control. Two cats were reportedly on an ectoparasite control product at the time of tick collection, including Revolution Plus (selamectin/sarolaner; *n* = 1) and Seresto collar (imidacloprid/flumethrin; *n* = 1). Five additional clinics reported that their patient had previously been prescribed ectoparasite control products including Revolution Plus (selamectin/sarolaner), Bravecto (fluralaner), Credelio (lotilaner), and Seresto (imidacloprid/flumethrin) but indicated that they were unsure of compliance or whether the products were given inconsistently. When asked about history of ectoparasite control, two clinics reported that owners had previously declined an ectoparasite control product but agreed to start an ectoparasite control product on the day of tick collection. No cat had a history of tick-borne disease diagnosis prior to the day of tick collection. Two cats presented with or developed clinical signs potentially related to tick infestation; one cat originating from Virginia was diagnosed with cytauxzoonosis on the day of tick collection and presented with numerous attached *I. scapularis*, severe lethargy, inappetence, icterus, and hypothermia. Cytology performed at the clinic revealed intra-erythrocytic piroplasms, presumed to be *C. felis*, and the cat was euthanized the same day owing to worsening condition. *I. scapularis* was the only species of tick submitted from this cat, and only one collected tick was found to be infected with *B. burgdorferi*. Another cat originating from Michigan developed an abscess at the site where the tick had been attached, a single *B. burgdorferi*-infected *I. scapularis*. Three additional cats had clinical signs at the time of tick collection but these were attributed to other ongoing conditions, including an abdominal mass (*n* = 1), chronic kidney disease (*n* = 1), and ear infection due to chronic polyps (*n* = 1).

## Discussion

In this study, we found that 12.0% of ticks collected from cats were infected with a tick-borne pathogen of zoonotic or feline health significance. This is lower than the previously reported 17.1% infection prevalence among ticks collected from cats in the USA [22]. In instances where multiple ticks of the same species were recovered from a single cat and tested positive for the same pathogen, it cannot be ruled out that the ticks shared the pathogen as a result of feeding in close proximity to one another, a phenomenon called co-feeding [37]. All pathogens detected in this study fell within previously reported geographic distributions [38–40]. In the present study, we found lower infection prevalence with *B. burgdorferi* and *A. phagocytophilum* among *I. scapularis*, 19.5% and 0.7% respectively, compared with what had been previously reported (25.7% and 4.4%) in *I. scapularis* collected from cats [22]. However, a greater proportion of *I. scapularis* tested in this study originated from areas outside the endemic distribution of *B. burgdorferi* and *A. phagocytophilum* compared with the previously reported survey, which may contribute to lower prevalence of these agents. We found very low prevalence of tick infection with *C. felis*. This finding is consistent with what has been previously reported in ticks collected from cats and wild-caught ticks in areas where cytauxzoonosis occurs [22, 34]. In this study, we detected *E. ewingii* in 3.2% of *A. americanum* and *B. miyamotoi* in 0.2% of *I. scapularis*. These pathogens were not detected in the previous survey of Ixodid ticks collected from cats in the USA [22]. *Ehrlichia ewingii* causes granulocytic ehrlichiosis in humans and dogs, primarily in central and southern USA [10, 41]. The agent has also been detected in a domestic cat with concurrent clinical signs, and a nationwide survey found 0.3% of cats were seroreactive to an *E. ewingii*-specific analyte [17]. *Borrelia miyamotoi* is transmitted by *I. scapularis* and overlaps in geographic distribution with Lyme disease [42]. *B. miyamotoi* is a relapsing fever *Borrelia* that can cause clinical disease in humans but is likely overshadowed by, and potentially misdiagnosed as, Lyme disease in areas where infection occurs [42]. *B. miyamotoi* has been previously detected in two asymptomatic cats [43], with little information available regarding infection prevalence or clinical significance in domestic cats.

In this study, approximately one in seven cats infested with ticks harbored at least one tick infected with a known pathogen of zoonotic or feline health significance. However, the clinical information collected here did not yield significant data on the relationship between exposure to tick-borne pathogens and development of clinical signs. An inherent limitation of this style of study is that detection of a pathogen within a tick does not imply pathogen transmission from the tick to the host nor subsequent development of disease if transmission did occur. Transmission of tick-borne pathogens is dynamic and depends on duration of tick attachment, host susceptibility, and how much infectious material is introduced to the host. Furthermore, a substantial amount of time elapsed between our contacting clinics and their original tick submission, which may affect the accuracy and availability of clinical information obtained. Only two cats had clinical signs concurrent with or within 1 month of tick collection that are likely related to tick infestation. In the case of the cat diagnosed and euthanized for cytauxzoonosis, transmission was presumably from exposure to *A. americanum* 11–13 days prior to presentation and discovery of infestation with *I. scapularis* [34, 44]. As expected, the vast majority of cats infested with pathogen-positive ticks had no history of ectoparasite control or known compliance failures in their ectoparasite control program. It is logical that this lack of ectoparasite control could be extrapolated to the entire feline population included in this survey. Two clinics reported that following tick removal, owners that had previously declined ectoparasite control agreed to start their cat on a tick-control product. Anecdotally, ectoparasites are particularly damaging to the human–animal bond because they are grossly visible. Furthermore, pet ownership has been associated with increased human and tick encounters in the home, increasing opportunities for tick-borne pathogen transmission [45].

In this study, ticks were collected from cats during all months of the year (Fig. 2). As expected, the diversity and density of ticks collected varied according to the seasonality of individual tick species and geographic location. Regardless, our data suggest that cats in all regions of the USA are at risk for tick infestation for at least a portion of the calendar year and may be at nearly continuous risk for tick infestation in regions where the environmental conditions support multiple tick species with complementary seasonalities. While the majority of cats in this study reportedly spent at least half of their time outside, 4.5% cats reportedly had no outdoor access but were infested with ticks. It is possible that time spent outdoors was misreported in these cases; however, it cannot be excluded that indoor-only cats may acquire ticks from other routes, such as other pets or people bringing ticks into the home. Although the clinical significance of many tick-borne zoonoses remains unclear in domestic cats, this study highlights the importance of compliant, continuous, and effective ectoparasite control for all cats regardless of lifestyle. Some feline tick control products have been demonstrated to effectively prevent transmission of tick-borne infections by killing ticks before transmission occurs [46, 47]. In the absence of adequate tick prevention, cats may be subject to direct injury caused by infestation, repeated exposure to tick-borne pathogens, and may transport pathogen-infected ticks in and around the home, which presents health concerns for other pets and humans in the vicinity.

## Conclusions

This study is the largest survey for tick-borne pathogens within Ixodid ticks collected from domestic cats in the USA. It is also the only survey of this kind in which retrospective contextual and clinical data were gathered to investigate the relationship between potential exposure to tick-borne pathogens and presence of clinical signs after exposure to pathogen-infected ticks. We found that ticks infesting cats may be infected with a variety of tick-borne pathogens, indicating potential for feline transmission and exposure to these infectious agents. Many of the pathogen-positive ticks in the present study were infected with agents of zoonotic importance. Our findings failed to establish a meaningful relationship between potential exposure to tick-borne pathogens and development of clinical signs among the cats included in the study population. Our data suggest that even indoor-only cats are not completely protected from tick infestation. Not only do ticks potentially pose a risk to feline health, but cats harboring ticks in and around the home also pose risk to other pets and people in the home. The importance of continuous, compliant ectoparasite control for cats, including ticks, cannot be overstated.

## Supplementary Information

Additional file 1. **Fig S1. **Geographic distribution of ixodid ticks collected from domestic cats presented to veterinary clinics.

Additional file 2.

Additional file 3.

## Data Availability

All the data generated in the paper are available in the paper itself.
