# Supplementary material for: Detection of pathogens within Ixodid ticks collected from domestic cats across the USA
Source: Parasit Vectors. 2025 Jul 4;18:255. doi: 10.1186/s13071-025-06902-z (PMC12228308; doi:10.1186/s13071-025-06902-z)
Supplement: Supplementary file 2 — Additional file 2. [file 13071_2025_6902_MOESM2_ESM.docx]

Table S1. PCR primers sequences and corresponding references used in this study

| **Target Organism(s)** | **Primers** | **Citations** |
| --- | --- | --- |
| *Borrelia* spp. | 1° FlaRL: 5’-GCAATCATAGCCATTGCAGATTGT-3’  FlaLL: 5’-ACATATTCAGATGCAGACAGAGGT-3’  2° FlaRS: 5’-CTTTGATCACTTATCATTCTAATAGC-3’  FlaLS: 5’-AACAGCTGAAGAGCTTGGAATG-3’ | (Barbour et al., 1996) |
| *Anaplasma phagocytophilum* | 1° ECB: 5’-CGTATTACCGCGGCTGCTGGCA-3’  ECC: 5’-AGAACGAACGCTGGCGGCAAGCC-3’  2° GE9F: 5’-AACGGATTATTCTTTATAGCTTGCT-3’  GA1UR: 5’-GAGTTTGCCGGGACTTCTTCT-3’ | (Little et al., 1997) |
| *Ehrlichia ewingii* | 1° ECB: 5’-CGTATTACCGCGGCTGCTGGCA-3’  ECC: 5’-AGAACGAACGCTGGCGGCAAGCC-3’  2° EE72: 5’-CAATTCCTAAATAGTCTCTGACTATT-3’  HE3: 5’-TATAGGTACCGTCATTATCTTCCCTAT-3’ | (Anderson et al., 1992; Dawson et al., 1996) |
| *Ehrlichia chaffeensis* | 1° ECB: 5’-CGTATTACCGCGGCTGCTGGCA-3’  ECC: 5’-AGAACGAACGCTGGCGGCAAGCC-3’  2° HE1: 5’-CAATTGCTTATAACCTTTTGGTTATAAAT-3’  HE3: 5’-TATAGGTACCGTCATTATCTTCCCTAT-3’ | (Anderson et al., 1992; Dawson et al., 1996) |
| *Cytauxzoon felis* | 1° C. felis I/IIF: 5’-AACCTGGTTGATCCTGCCAGTAGTCATATGCTTG-3’  C. felis IIR: 5’-TCACCAGAAAAAGCCACAAC-3’  2° CfnestF: 5’- TCGCATTGCTTTATGCTGGCGATG-3’  CfnestR: 5’- GCCCTCCAATTGATACTCCGGAAA-3’ | (Bondy et al., 2005; Reichard et al., 2010) |
| *Rickettsia* spp. | 1° 17K-5: 5’-GCTTTACAAAATTCTAAAAACCATATA-3’  17K-3: 5’-TGTCTATCAATTCACAACTTGCC-3’  2° 17K-1: 5’-GTCCTTGCAACTTCTATGTT-3’  17K-2: 5’-CATTGTTCGTCAGGTTGGCG-3’ | (Stothard, 1995; Heise et al., 2010) |
