# Supplementary material for: Detection of pathogens within Ixodid ticks collected from domestic cats across the USA
Source: Parasit Vectors. 2025 Jul 4;18:255. doi: 10.1186/s13071-025-06902-z (PMC12228308; doi:10.1186/s13071-025-06902-z)
Supplement: Supplementary file 3 — Additional file 3. [file 13071_2025_6902_MOESM3_ESM.docx]

Table 2. Categorically summarized responses received from clinics regarding the ectoparasite prevention history, clinical status, and history of tick-borne disease from domestic cats with pathogen-positive ticks

| **Question provided to clinic** | **Responses** |
| --- | --- |
| At the time of tick collection, was the patient being prescribed an ectoparasite control product? | Yes (n=2)  Inconsistent use or suspected non-compliance (n=5)  No (n=25)  Unknown (n=4) |
| Has the patient ever been previously diagnosed with a tick-borne disease? | No (n=34)  Unknown (n=2) |
| Did the patient have any overt clinical signs at the time of or shortly following (within 1 month) of tick collection which could be attributable to tick borne disease (including but not limited to fever, lameness, blood abnormalities)? | Clinical signs potentially related to tick or tick-borne disease (n=2)  Clinical signs related to other known condition (n=3)  No apparent clinical signs (n=30)  Unknown (n=1) |
